# Intravesical gemcitabine versus mitomycin for non-muscle invasive bladder cancer: a systematic review and meta-analysis of randomized controlled trial

**DOI:** 10.1186/s12894-020-00610-9

**Published:** 2020-07-13

**Authors:** Rongxin Li, Ye Li, Jun Song, Ke Gao, Kangning Chen, Xiaogang Yang, Yongqiang Ding, Xinlong Ma, Yang Wang, Weipeng Li, Yanan Wang, Zhiping Wang, Zhilong Dong

**Affiliations:** 1 Lanzhou University Second Hospital, Lanzhou City, Gansu Province China; 2 Sanya People’s Hospital, Sanya City, Hainan Province China

**Keywords:** Bladder cancer, Gemcitabine, Mitomycin, Systematic evaluation, Meta-analysis

## Abstract

**Background:**

Mitomycin (MMC) has been frequently used as the compound for intravesical treatment. The relatively new pyrimidine analog gemcitabine (GEM) has exhibited anticancer effect on various solid cancers, such as the advanced bladder cancer. In this study, the GEM and MMC in treating non-muscle invasive bladder cancer (NMIBC) cases was compared through systemic review.

**Methods:**

In accordance with the Preferred Reporting Items for Systematic Reviews and Meta-Analyses (PRISMA) statement, the electronic databases, including Embase, PubMed, Chinese biomedicine literature database, the Cochrane Library, the National Institute for Health and Clinical Excellence, NHS Evidence, Chinese technological periodical full-text database, and Chinese periodical full-text database, were systemically reviewed from inception to October 2018. Then, the RevMan 5.0 software was applied for data analysis. Five randomized controlled trials (RCTs) involving a total of 335 patients were included.

**Results:**

For MMC group, the recurrence rate in the mitomycin arm increased compared with that in GEM group (OR = 0.44 95% CI [0.24, 0.78]), and the difference was statistically significant between the two groups. GEM was associated with reduced incidence of chemical cystitis compared with that of MMC (OR = 0.23 95% CI [0.12, 0.44]). Differences in hematuria (OR = 0.46 95% CI [0.16, 1.31]), skin reaction (OR = 0.49 95% CI [0.14, 1.70]) and liver and kidney function damage (OR = 0.51 95% CI [0.09, 2.85]) displayed no statistical significance between the two groups.

**Conclusion:**

Findings in our study demonstrate the superior efficacy of GEM over MMC in reducing the relapse rate among NMIBC patients following transurethral resection (TUR). In addition, GEM is associated with reduced local toxic effects on the bladder compared with those of MMC. However, more future studies are needed to examine GEM safety when used as the monotherapy or polytherapy for bladder patients. More RCTs with high quality are also required to validate our findings due to the limitations of the current meta-analysis.

## Background

Bladder cancer has become a common cancer worldwide, and 430,000 new cases and over 165,000 deaths are reported in 2012 [1]. Transitional cell carcinoma is dominant in bladder cancer, in addition, adenocarcinoma (cancer originating from the mucus-making and releasing cells) and squamous cell carcinoma (cancer originating from the thin and flat cells) are also observed [2]. Generally, bladder cancer is associated with the following symptoms, frequent urination, bloody urine (rendering the slight rusty to deep red color of urine), urination pain, or urination sensation with no urine indeed [3].

The incidences of bladder cancer in male and female are reported to be 3.4 and 1.2%, respectively, however, that in patients aged over 70 years is twice to thrice higher than that among patients aged 55–65 years, and 15 to 20 times greater than for patients aged 30–54 years [4]. As estimated by the World Health Organization, there were 132,432 bladder cancer-related deaths in the world in 2000 [5].

About 80% bladder cancers are non-muscle invasive bladder cancers (NMIBC) restricted to the urothelium (clinical stage Ta) or lamina propria (stage T1) at first, which is featured by the in-situ flat carcinoma (stage Tis) [6]. Tumor relapse following transurethral resection (TUR) has been identified as a main issue in treating non-muscle invasive bladder cancer. Specifically, the mechanisms regarding NMIBC relapse following TUR are shown as follows, (1) residual tumor originated from the incomplete resection; (2) floating cancer cell implantation in traumatized bladder sites; (3) incidence of new neoplasm due to high cancer aggressiveness; (4) relapses at the urothelial instability sites (atypia, hyperplasia and dysplasia) [7]; (5) over 50–70% tumor relapse since no adjuvant treatment is given, and approximately 15% cases develop muscle-invasive cancers eventually [8].

The intravesical chemotherapy has been considered as the standard treatment for patients receiving following TUR to eradicate underlying disorders, inhibit cancer relapse, prevent tumor development and extend patient survival [9]. Mitomycin (MMC) is a frequently used compound in intravesical treatment. In addition, gemcitabine (GEM), the relatively new pyrimidine analog, displays anticancer effect on various solid cancers, such as the advanced bladder cancer [10]. MMC and GEM have been classically used as the cytotoxic agents for affecting the DNA integrity, but these two display distinctly different mechanisms of action. Among them, GEM, the difluoro-2, 2-deoxy-cytidine, can be activated upon the stimulation of deoxycytidine kinase, meanwhile, its phosphorylated metabolites may impact the synthesis of deoxy-nucleotide through resulting in DNA injuries and interfering with DNA repair [11]. On the other hand, MMC can be triggered within cancer cells through forming the reducing equivalents, thereby affecting cancer cell replication through forming the damage-induced DNA adducts [12]. Nonetheless, it remains unclear about the efficacy and side effects between the two agents. In this regard, the current systematic review was carried out aiming to evaluate the therapeutic effects and safety between GEM versus MMC on treating NMIBC patients.

## Methods

### Search strategy

In accordance with Preferred Reporting Items for Systematic Reviews and Meta-Analyses (PRISMA) statement [13], the electronic databases, including Embase, PubMed, Chinese biomedicine literature database, the Cochrane Library, the National Institute for Health and Clinical Excellence, NHS Evidence, Chinese periodical full-text database, and Chinese technological periodical full-text database, were systemically reviewed from inception to October 2018 to identify the randomized controlled trials (RCTs) that compared the efficacy and safety of GEM and MMC in treating NMIBC cases. Additionally, the critical Chinese magazines in relevant fields were also retrieved manually, corresponding search engines were employed to identify the relevant references, and each reference in the enrolled articles was retrieved as well to discover other possible relevant publications. Related terms were used for study retrieval. Moreover, the reference lists from those enrolled articles and reviews were also manually retrieved, and experts in this field were contacted if necessary, while those non-published articles were not found. The study retrieval was not restricted by language.

In this study, the following search terms were used in retrieval strategy, including intravesical pharmacotherapy, non-muscle invasive bladder cancer, gemcitabine and mitomycin, so as to find out the titles and abstracts of relevant studies. To be specific, the entire retrieval strategy adopted in the current work for the PubMed database included (irrigation of bladder OR intravesical therapy OR bladder instillation OR intravesical instillation OR intravesical infusion OR infusion of bladder) AND (non-muscle-invasive bladder cancer OR NMIBC OR non-muscle invasive bladder cancer OR superficial bladder cancer) AND (GEM OR gemcitabine) AND (MMC OR mitomycin OR mitomycin-c).

### Data collection

Two researchers independently reviewed those retrieved titles and abstracts in accordance with the study inclusion criteria, and the inappropriate articles were excluded. Any disagreement between them was settled down through the opinion from a third author. Afterwards, data were extracted by the same authors independently by the use of the uniform data collection forms. Besides, the evaluated quality items included the concealment of allocation, randomization, blinding (subjects, researchers, outcome measures, data analysis), as well as follow-up completeness [14].

### Inclusion and exclusion criteria

The study inclusion criteria were shown as follows: (1) randomized controlled trials (RCTs) as well as quasi-RCTs (namely, RCTs adopting the quasi-random method for participating allocation into various intervention groups); (2) studies that comprised medium- to high-risk patients (with high-grade papillary stage Ta or T1 tumors and any patient with carcinoma in situ) who occupies 15–44% NMIBC cases in certain series [15]; (3) studies that mentioned clinical outcomes, Ta or T1 tumor and included patients receiving intravesical gemcitabine comparing with mitomycin.

The study exclusion criteria were as follows: (1) non-RCTs; (2) studies in which cases with other neoplasm; (3) studies with incomplete information to analysis; (4) duplicate articles.

### Outcome measure types

Tumor relapse rate, and local side effects (including chemical cystitis, hematuria, skin reaction, and liver and kidney function damage).

### Intervention types

Intravesical treatment with GEM or MMC following TUR.

### Statistical analysis

All dichotomous outcomes (such as recurrence, mortality, progression of tumor staging, distant metastasis, systemic and local adverse reaction, treatment delay or withdrawal) were presented in the manner of relative risk (RR) and the corresponding 95% confidence intervals (CI). The data were extracted and pooled using the random-effects model. For guaranteeing our model robustness and outlier susceptibility, we also adopted the fixed-effect model for analysis. The statistical heterogeneities across different trials were tested using the chi-square heterogeneity test while the inconsistency degree was assessed by I^2^ statistic, with the threshold of *p* = 0.10, and *p* < 0.1 indicated the presence of statistical heterogeneity across various studies. Thereafter, subgroup analyses, sensitivity analysis, and the random effects model were carried out in the presence of heterogeneity. The potential heterogeneity sources were explored through subgroup analysis. The putative factor impacts on the effect size were explored through sensitivity analysis. The descriptive approaches were adopted for tabulating and assessing the adverse effects, as they were possibly distinct for our examined agents.

## Results

### Search results

**Fig. 1:**
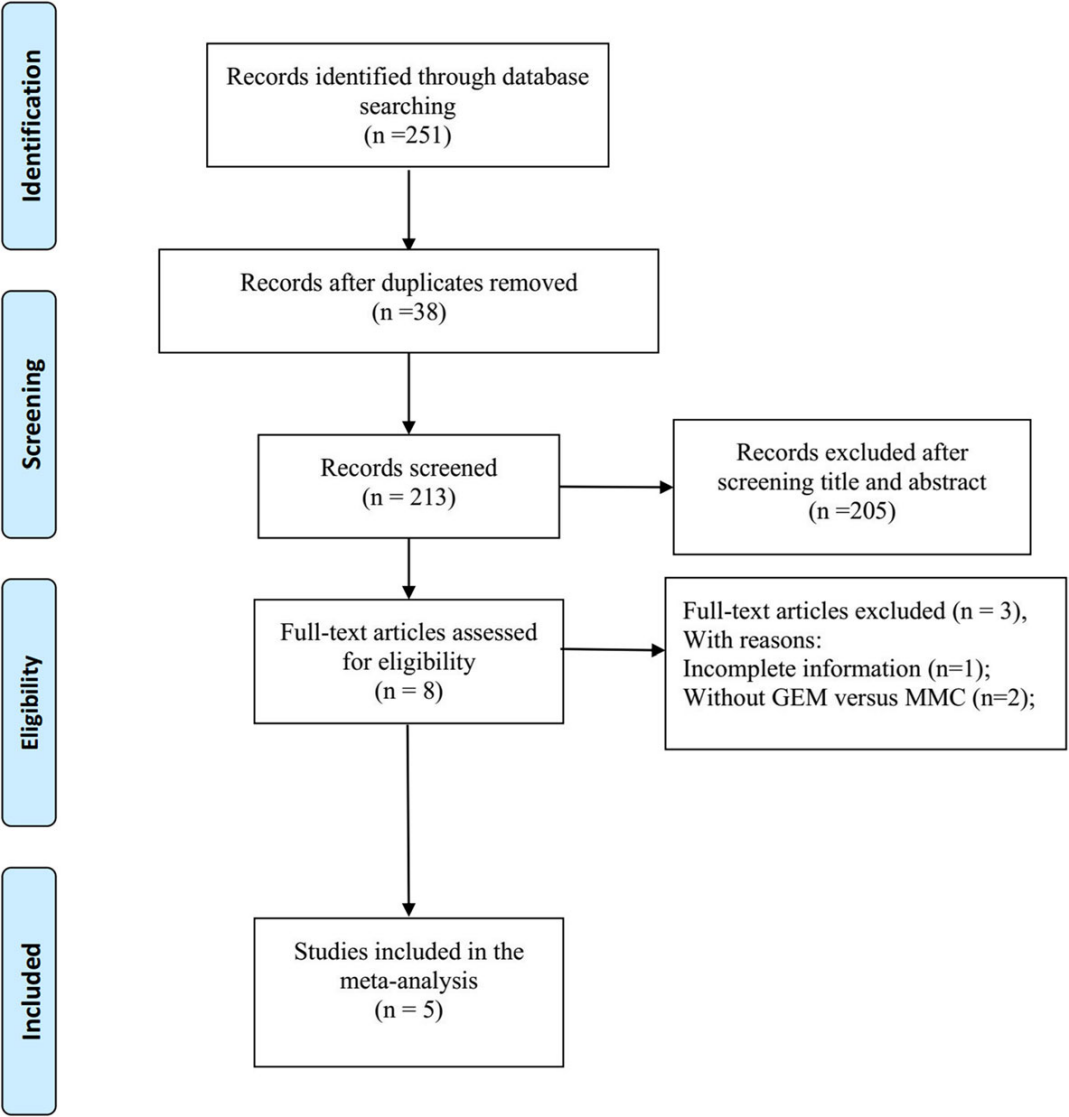
PRISMA flow diagram: the study selection process

Figure 1 displays the study retrieval and screening procedure. A total of 213 preliminary studies were retrieved initially, among which, 208 were ruled out from this study later. Finally, 5 RCTs including 335 cases were included [16–20], all of which mentioned outcomes including recurrence rate and toxicity evaluation (chemical cystitis, hematuria, skin reaction, and liver and kidney function damage).

### Included study features and quality

Table 1 displays the enrolled study features and quality. Table 2 presents the patient features and drug administration schedule.

**Table 1:** The quality and characteristics of included studies

Study	Randomization	Allocated Concealment	Blinding	Gemcitabine (sample size)	Mitomycin (sample size)	Grade
Dong 2017 [16]	adequate	not used	clear	12	16	**C**
Lin 2016 [17]	adequate	not used	clear	42	42	**C**
Sun 2016 [18]	adequate	used	clear	30	28	**B**
Xiaohong 2015 [19]	adequate	not used	clear	27	29	**C**
Raffaele 2010 [20]	adequate	used	clear	54	55	**B**

**Table 2:** Characteristics of studies and the schedule of drug administration among the included studies

Study	Cases	Sex(M/F)	Age (year)	Type of patients	Stage	Grade	Comparison	Dosage of intervention	Follow-up
Dong 2017 [16]	28	NA	NA	NMIBC	NA	NA	GEM	1000 mg weekly for 8 weeks followed by monthly instillations	12 months
							MMC	20 mg weekly for 8 weeks followed by monthly instillations	
Lin 2016 [17]	84	56/28	GEM:61 ± 4.2 MMC:62 ± 3.5	NMIBC	Tis: 3 Ta: 24 T1: 59	G1: 40 G2: 27 G3: 17	GEM	1000 mg weekly for 8 weeks followed by monthly instillations (a total of 1 year or 2 years)	24 months
							MMC	30 mg weekly for 8 weeks followed by monthly instillations (a total of 1 year or 2 years)	
Sun 2016 [18]	58	45/13	GEM: 37 ~ 83 MMC: 31 ~ 81	NMIBC	NA	NA	GEM	1000 mg weekly for 8 weeks followed by 10 monthly instillations (a total of 1 year)	2 years
							MMC	20 mg weekly for 8 weeks followed by 10 monthly instillations (a total of 1 year)	
Xiaohong 2015 [19]	56	45/11	GEM:51.26 ± 13.42 MMC:51.64 ± 12.39	NMIBC	Tis: 1 Ta: 10 T1: 45	G1: 13 G2: 41 G3: 2	GEM	1000 mg weekly for 8 weeks followed by 10 monthly instillations (a total of 1 year)	1 year
							MMC	40 mg weekly for 8 weeks followed by 10 monthly instillations (a total of 1 year)	
Raffaele 2010 [20]	109	93/16	GEM: 64.9 ± 10.5 MMC: 67.9 ± 10.2	NMIBC	Ta: 72 T1: 37	G1: 25 G2: 55 G3: 29	GEM	2000 mg weekly for 6 weeks followed by 10 monthly infusion during the first year	36 months
							MMC	40 mg weekly for 4 weeks followed by 10 monthly infusion during the first year	

*M* Male, *F* Female, *NMIBC* Non-muscle invasive bladder cancer, *GEM* Gemcitabine, *MMC* Mitomycin, *NA* Not Available (Insufficient Information Provided)

### Meta-analysis results

#### Treatment efficacy

**Fig. 2:**
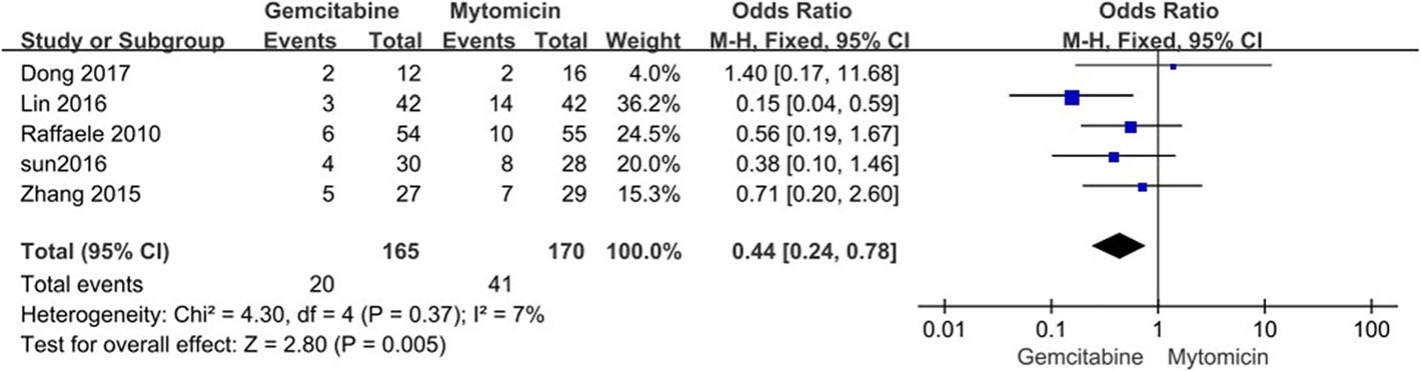
Recurrence rates: gemcitabine versus mitomycin

**Fig. 3:**
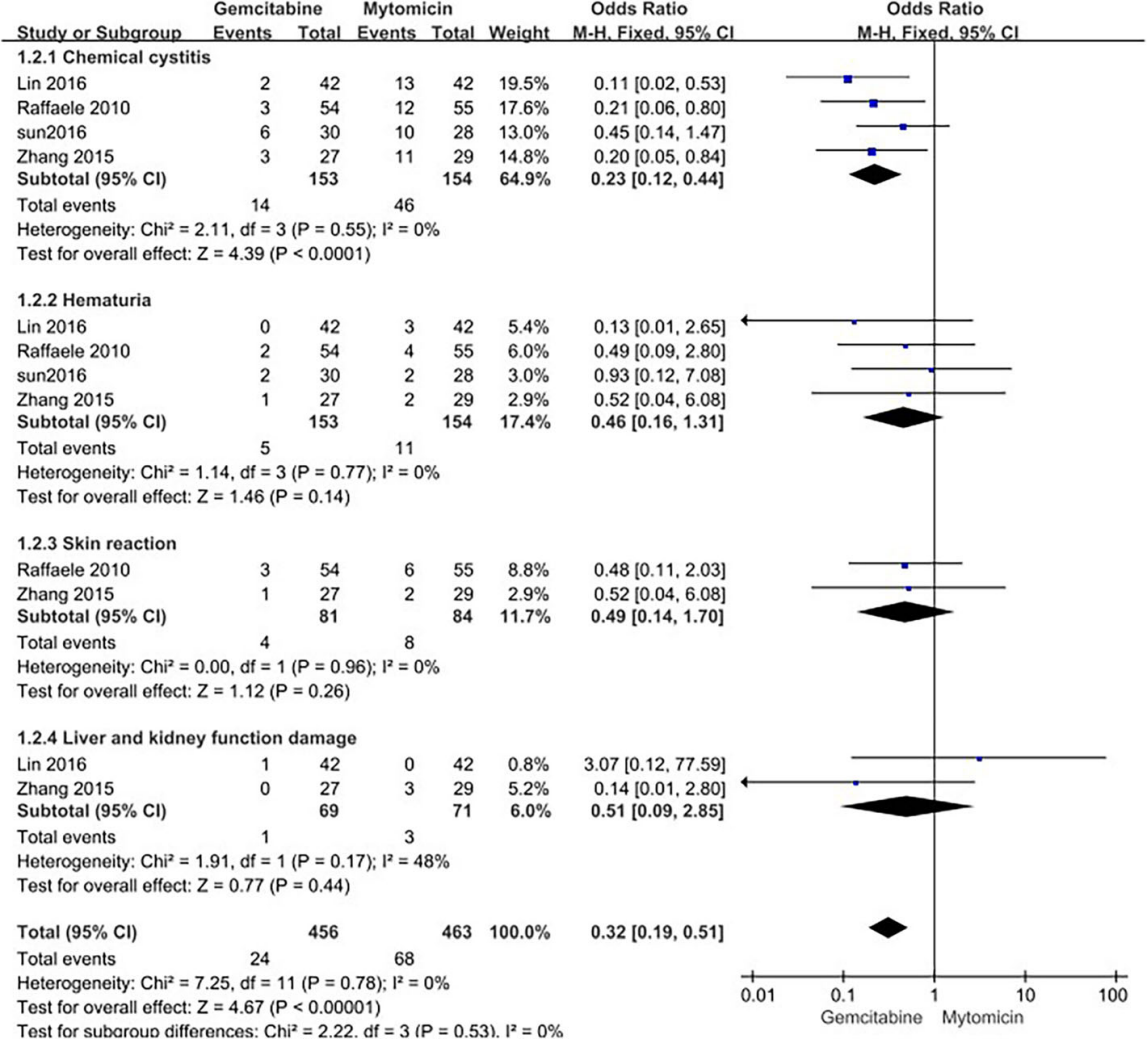
Local side effects: gemcitabine versus mitomycin

For MMC group, the recurrence rate increased compared with that of GEM group (OR = 0.44 95% CI [0.24, 0.78]), and the difference was of statistical significance between the two groups, as shown in Figs. 2 and 3.

#### Adverse effects

Five trials reported the local adverse effects. The most commonly reported adverse effects in both groups were chemical cystitis (OR = 0.23 95% CI [0.12, 0.44]), hematuria (OR = 0.46 95% CI [0.16, 1.31]), skin reaction (OR = 0.49 95% CI [0.14, 1.70]) and liver and kidney function damage (OR = 0.51 95% CI [0.09, 2.85]). According to our results, GEM was associated with reduced local toxicity relative to mitomycin. Overall, these two doses were well tolerable among a majority of cases with reduced adverse reactions.

## Discussion

Meta-analysis is an approach adopted to statistically pooled and examined the findings from several independent RCTs [21]. NMIBC is generally associated with favorable clinical outcomes, but it is refractory due to the increased tumor relapse rate following TUR. As a matter of fact, 40–85% cases develop tumor relapse in 2–5 years following standardized TUR, while approximately 10% among such relapse patients are at the more advanced grade and stage [22]. In clinical practice, TUR is performed prior to intravesical injection with chemotherapeutic or the agents for modulating immunity, so as to lower the bladder cancer relapse and development.

Various anti-cancer agents are administered intravesically to prevent tumor relapse, which is validated as effective. The classical intravesical agents, Bacille Calmette-Guérin (BCG) as well as MMC are effective on postponing and lowering the postoperative tumor relapse rate; nonetheless, they are also associated with obvious adverse reactions and may be ineffective for some cases. Based on clinical research, GEM is effective on reducing the relapse of non-muscle invasive bladder cancer with lower toxicity, which deserves more investigation [23]. In this study, the meta-analysis was carried out to examine the intravesical GEM and MMC safety and efficacy among the NMIBC patients on the basis of the five enrolled clinical trials including 335 bladder cancer cases. Three of those 5 articles mentioned disease grade and stage [17, 19, 20]. Our results showed that the recurrence rate of MMC group increased compared with that of GEM group (OR = 0.44 95% CI [0.24, 0.78]), and the difference was statistically significant between the two groups; besides, GEM led to a lower incidence of chemical cystitis than MMC (OR = 0.23 95% CI [0.12, 0.44]). But differences in the incidences of hematuria (OR = 0.46 95% CI [0.16, 1.31]), skin reaction (OR = 0.49 95% CI [0.14, 1.70]) and liver and kidney function damage (OR = 0.51 95% CI [0.09, 2.85]) were not statistically significant between the two groups., suggesting that GEM had lower toxicity than MMC. Overall, these two doses were well-tolerable among a majority of cases with lower adverse reactions. Nonetheless, the above conclusions must be interpreted carefully and validated in more articles.

For the low risk disorder, post-operative intravesical MMC treatment is still the vital part of intravesical therapy, meanwhile, there is level-one evidence to prove the effectiveness of GEM on bladder cancer, yet it has not been recommended in guidelines [24]. Many therapeutic methods are effective on the treatment of NMIBC cases. The intravesical chemotherapy using MMC and GEM has achieved certain favorable results [25]. Gemcitabine is an effective pyrimidine antimetabolite, with only mild toxicity relative to other chemotherapeutic agents [26]. According to Messing EM et al. [27], for the low-grade NMIBC cases (*n* = 215), those receiving intravesical GEM treatment following TUR were associated with the 4-year tumor relapse rate of 34%, while that in the saline-alone control group was 54%. In addition, 5 cases of GEM group and 10 in control group progressed to muscle invasive bladder cancer, and 17 of GEM group as well as 25 of saline group died. Based on Dalbagni et al., for the 30 BCG-refractory NMIBC cases (including uncontrollable Ta, multiple unresected T1, and refractory cancer in situ) who underwent intravesical GEM treatment regularly, 50% achieved a complete response (CR), and adverse effects were reported among 23% cases with a very low toxicity [28]. Moreover, according to Bartoletti et al., no 1-year tumor relapse was reported among 18 out of the 24 medium-risk patients, as well as among 9 out of the 16 high-risk BCG-refractory cases (pTa ~ pT1) treated with weekly intravesical GEM therapy. In that study, no side effect was reported in 94 of the 116 patients in the 1-year treatment process [29]. Jones et al. reviewed 6 GEM treatment-related RCTs in their Cochrane review, and indicated that, intravesical GEM treatment potentially exerted an important part in managing NMIBC cases with moderate and high risk, particularly when it was adopted to be the alternative to MMC for high-risk cases [30].

Auxiliary intravesical treatment can lower the relapse and development rates of NMIBC in the meantime of inducing local and systemic adverse reactions. In terms of the toxic effects, chemical cystitis, hematuria, skin reaction, and liver and kidney function damage were observed in 0–30% cases of each group. The frequency of these side effects of GEM arm markedly decreased compared with that of MMC arm. Such findings validated that, chemical cystitis, the irritable symptom in lower urinary tract, stands for the typical MMC side reaction [31]. GEM led to minimal local toxicity, which was self-resolved rapidly. By contrast, MMC treatment resulted in the more serious local toxicity that required treatment delay. On the other hand, economic influence is also a factor potentially affecting the treatment decision-making. Recently, the mean sales price for 40 mg MMC markedly increases compared with that for 2 g GEM, suggesting that the GEM-based intravesical chemotherapy is advantageous over MMC in price [32].

Some limitations should be noted in the current meta-analysis: 1) the risk of tumor relapse was not compared between GEM and MMC of Ta or T1 group due to the limited total sample size, so more high-quality RCTs should be carried out. 2) The administration does and schedules of these two agents were similar among those five enrolled studies, but the conclusion might be disturbed by the clinical heterogeneity because of the differences in operating methods, like whether the positional changes were allowed, as well as the duration of each infusion. 3) All included RCTs in the current review did not apply the double-blinding method, which might potentially impact our results. 4) The publication bias was not totally ruled out, which possibly led to conclusion distortion.

## Conclusions

In conclusion, our meta-analysis supports that GEM can be a potential agent of intravesical therapy with better efficiency than MMC in preventing recurrence of patients with non-muscle invasive bladder cancer after TUR. At the same time, we observed that GEM produce lower local toxic effects than MMC during intravesical therapy. In addition, more studies are needed to examine the GEM safety, and more high-quality RCTs are warranted to validate our conclusions due to the limitations.

## Data Availability

The datasets used and/or analyzed during the current study are available from the corresponding author on reasonable request.

## Abbreviations

BCG: Bacille Calmette-Guérin
CI: Confidence intervals
GEM: Gemcitabine
MMC: Mitomycin C
NMIBC: Non-muscle-invasive bladder cancers
PRISMA: Preferred Reporting Items for Systematic Reviews and Meta-Analyses
RCTs: Randomized controlled trials
TUR: Transurethral resection
